# Impact of 3D Rigid Ring Annuloplasty for Tricuspid Regurgitation: A Systematic Review and Meta-Analysis

**DOI:** 10.3389/fcvm.2022.725968

**Published:** 2022-03-08

**Authors:** Tao You, Yu-Hu Ma, Kang Yi, Jie Gao, Jian-Guo Xu, Xiao-Min Xu, Shao-E He, Wei Wang, Meng Ji

**Affiliations:** ^1^Department of Cardiovascular Surgery, Gansu Provincial Hospital, Lanzhou, China; ^2^Gansu International Scientific and Technological Cooperation Base of Diagnosis and Treatment of Congenital Heart Disease, Lanzhou, China; ^3^Department of Neurosurgery, West China Hospital of Sichuan University, Chengdu, China; ^4^The First Clinical Medical College of Lanzhou University, Lanzhou, China; ^5^Evidence-Based Medicine Center, School of Basic Medical Sciences, Lanzhou University, Lanzhou, China; ^6^The Second Clinical Medical College of Lanzhou University, Lanzhou, China

**Keywords:** tricuspid regurgitation, three-dimensional rigid ring, tricuspid annuloplasty, meta-analysis, systematic review

## Abstract

**Background:**

Tricuspid annuloplasty (TAP) is accepted as the standard technique for correcting tricuspid regurgitation (TR). We conducted the present study to provide an overview of the contemporary results of 3D rigid ring annuloplasty for TR.

**Methods:**

A systematic literature search was carried out in eight databases to collect all relevant studies on the three-dimensional (3D) rigid ring annuloplasty treatment of TR published before October 1, 2020. The main outcomes of interest were postoperative TR grade, perioperative mortality, and recurrent TR.

**Results:**

A total of eight studies were included, all of which were retrospective observational studies. Rigid 3D rings were compared with flexible bands, and there was no difference in perioperative mortality [odds ratio (*OR*) = 1.02; 95% *CI* (0.52, 2.02); *p* = 0.95], late mortality [*OR* = 0.99; 95% *CI* (0.28, 3.50); *p* = 0.98], or recurrent TR [*OR* = 0.59; 95% *CI* (0.29, 1.21); *p* = 0.15]. The postoperative TR grade associated with 3D rigid rings was 0.12 lower [mean difference (MD) = −0.12; 95% *CI* (−0.22, −0.01); *p* = 0.03], which indicated that 3D rigid rings result in better postoperative outcomes than flexible bands. Compared with suture annuloplasty, the postoperative TR grade of the 3D rigid ring group was 0.51 lower [MD = −0.51; 95% *CI* (−0.59, −0.43); *p* < 0.05]. Within the 5 years of follow-up, patients who underwent 3D rigid ring annuloplasty had lower TR recurrence [*OR* = 0.26; 95% *CI* (0.13, 0.50); *p* < 0.05].

**Conclusions:**

Compared with suture annuloplasty, 3D rigid rings present early advantages. The 3D rigid rings provide an acceptable short-term effect similar to that of the flexible bands, and a significant difference between these approaches was not discovered. However, the conclusion was based on the limited, short-term data available at the time of the study. Further research on the long-term effects of 3D rigid ring annuloplasty for TR is clearly needed.

**Systematic Review Registration:**

https://inplasy.com/inplasy-2021-3-0105/, identifier: 202130105.

## Introduction

Tricuspid regurgitation (TR) is a common valvular heart disease (VHD) that occurs in 65–85% of the population (1). Mild TR with a normal structure can be regarded as a normal variant. Moderate to severe TR is usually pathological (2) and is an independent risk factor for progressive heart failure and increased mortality (3–6). TR is divided into primary TR and functional TR (FTR). FTR is caused by the abnormal anatomy and function of the tricuspid valve due to dilation and dysfunction of the right ventricle. It is usually secondary to left heart disease, such as mitral regurgitation, mitral stenosis, and aortic stenosis (7). A total of 30–50% of patients with severe mitral regurgitation have obvious (moderate and severe) TR (8), and the incidence of TR in patients with severe aortic stenosis also exceeds 25% (9). In addition, atrial fibrillation may be an important cause of TR. In the absence of pulmonary hypertension or left-side heart disease, isolated TR can appear in elderly patients with a high incidence of atrial fibrillation (10–12). At present, FTR is considered to be a continuous process. If it is not treated, disease progression will lead to gradual dilation and dysfunction of the right ventricle, which will seriously affect the prognosis (13–15).

The American Heart Association (AHA) and European Society of Cardiology (ESC) guidelines recommend that patients with severe TR should be treated with the tricuspid valve at the same time as left heart valve surgery (Class I recommendation). For patients with mild to moderate TR and tricuspid annulus dilation, tricuspid valve surgery should be considered during the same period of left heart valve surgery (Class IIa recommendation) (16, 17). At present, transcatheter tricuspid valve intervention (TTVI) is developing rapidly, but the technology has not been fully popularized in clinical practice. Tricuspid valve plasty (TVP) is still the main method of surgical treatment of tricuspid regurgitation, mainly including suture annuloplasty and prosthetic tricuspid annuloplasty (18–20). Suture annuloplasty, such as the Kay method (21) and De Vega method (22), has the advantages of simple technology and low patient economic burden but also has a relatively high recurrence rate (23, 24). Compared with sutures, prosthetic tricuspid annuloplasty can better prevent annular dilatation, right ventricular volume overload, and right heart failure (25). Currently, a large number of studies have shown that the ability of prosthetic tricuspid annuloplasty to restore the tricuspid valve is better than that of suture annuloplasty, so tricuspid annuloplasty (TAP) using various commercially available rings is accepted as the standard technique for correcting TR (26–28).

According to the rigidity, TAP rings are divided into flexible bands and rigid rings. The former can adapt to the cyclical movement of the heart, whereas it cannot be maintained for a long time. Long-term right ventricular hypertension and valve movement will gradually expand the annulus and produce regurgitation. The latter is not well-adapted to the anatomical characteristics of the tricuspid valve annulus but maintains a relatively long time (29). Due to the unique dynamic three-dimensional (3D) structure of the tricuspid valve, many 3D rigid rings have been developed in recent years. It is believed that the 3D rigid ring can adapt well to the anatomical structure of the tricuspid valve, correct the expansion of the annulus, and prevent further expansion of the annulus (30). Studies have pointed out that it can enhance the joint force of the valve leaflets and reduce the tension of the suture, thereby reducing the possibility of long-term recurrence of tricuspid regurgitation (31).

In the past few decades, the surgical results of many types of annuloplasty have been reported clinically, but only a few studies have compared and evaluated these devices (32, 33). Therefore, it is still inconclusive which tricuspid annuloplasty ring should be chosen in clinical practice. Although the 3D rigid ring has been widely used in clinical practice, there is no relevant research to systematically explain whether it has advantages compared with suture, flexible band, and standard rigid ring TAP. Based on this, we conducted this systematic review and meta-analysis to compare the effects of 3D rigid annulus and other methods in TAP and provide a reference for selecting the appropriate annulus type during tricuspid annuloplasty.

## Materials and Methods

### Protocol and Registration

This systematic review and meta-analysis was performed according to the preferred reporting items for the systematic reviews and meta-analyses (PRISMA) statement. The protocol was registered on INPLASY (202130105) and is available in full on the inplasy.com (https://inplasy.com/inplasy-2021-3-0105). Ethical approval was not required for this work because this was an analysis of previously published data.

### Literature Search

**Figure 1:**
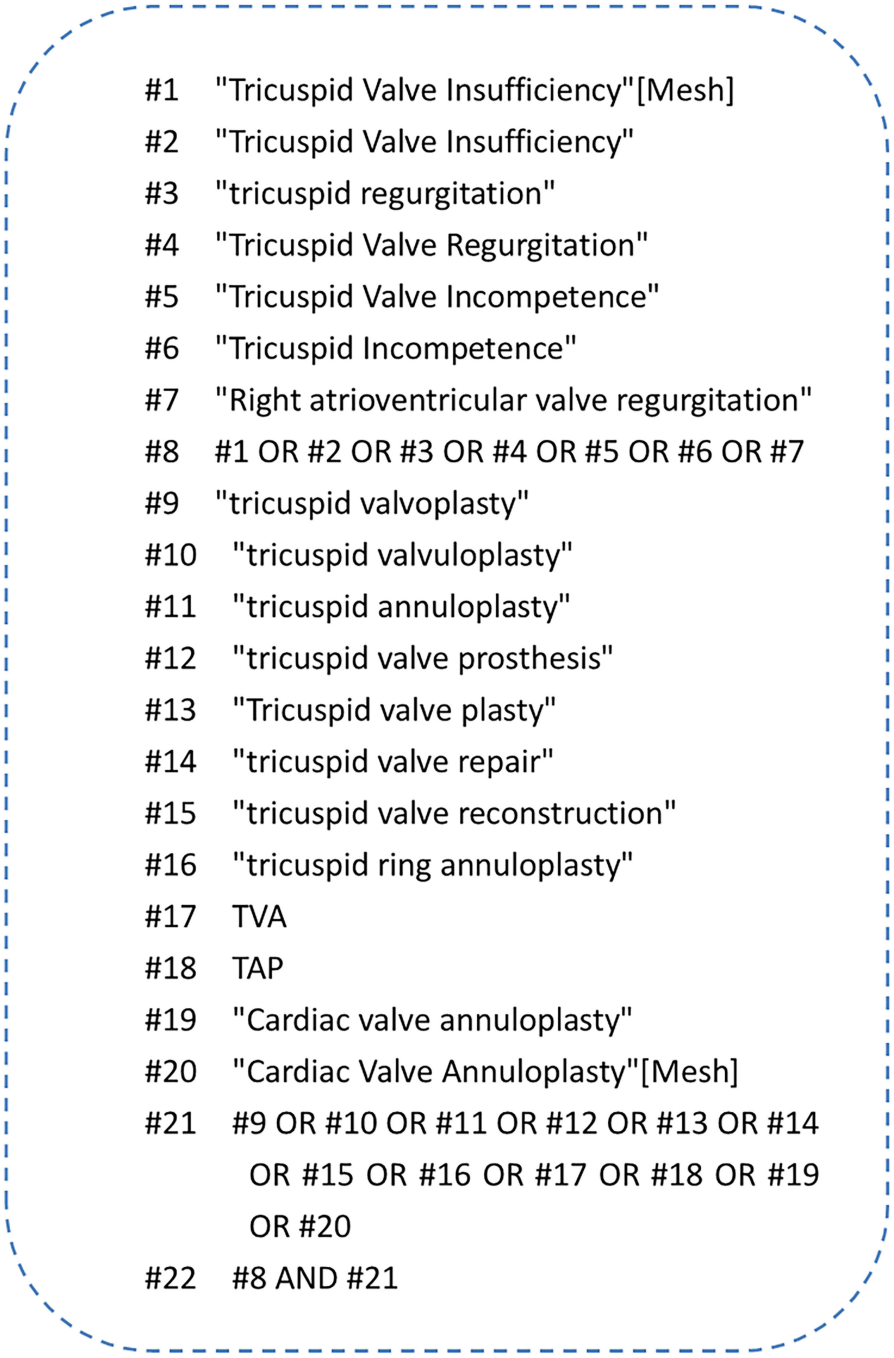
Search strategies for this study in PubMed.

We conducted a systematic literature search on eight databases, such as PubMed, the Cochrane Library, Web of Science, EMBASE, China National Knowledge Infrastructure (CNKI), China Biology Medicine disc (CBM), Wan Fang, and VIP, to retrieve all related articles before October 1, 2020. At the same time, we traced the references of the included literature and found documents through Google Scholar and manual search of related articles. Similarly, we searched the references of the included literature through the snowball method to maximize the sensitivity of retrieval as much as possible. Taking PubMed as an example, the specific retrieval strategy is shown in Figure 1.

### Inclusion and Exclusion Criteria

The inclusion criteria of this study were determined before the literature search. The included studies met the following inclusion criteria: (1) comparison of 3D rigid rings and sutures, flexible bands, flat rigid rings, and other shaping techniques in the treatment of TR, (2) randomized Controlled Trial (RCT) or cohort study, and (3) if several studies conducted by the same institution have overlapping samples, only the latest research literature will be included.

The exclusion criteria were as follows: (1) TR surgery with no direct comparison between 3D rigid ring and other plastic surgeries, (2) report on the combination of different forming technologies, (3) unable to obtain surgical information and other outcome indicators, (4) reviews, comments, letters, expert opinions, and case reports, and (5) animal-based studies.

### Data Extraction

Two reviewers (Yu-Hu Ma and Kang Yi) independently screened the literature according to the inclusion and exclusion criteria and cross-checked the literature. By reading the titles and abstracts of the obtained literature, trials that did not meet the inclusion criteria were excluded, and then, the full text of the suspected literature was read through to determine whether the study was included. If disagreement arose, the two people first had a discussion. If disagreement still existed, a third reviewer (Tao You) read the full text and participated in the discussion until an agreement was reached. According to the developed data extraction table, the data were extracted by using Microsoft Office Excel 2019. The extracted content mainly included the following. (1) Basic information of the included research was used, such as the first author, the year of publication, the country and region of the research object, and the duration of the research. (2) Basic information of patients was used, such as the follow-up time, sample size, type of forming ring, age, heart function grade, and tricuspid regurgitation grade of the two groups. (3) Outcome indicators included aortic cross-clamp (ACC) time, cardiopulmonary bypass (CPB) time, post-operative TR grade (defined as TR grade within 1 week after surgery), perioperative mortality (defined as hospital mortality or 30-day death rate), late mortality rate (defined as the total mortality rate during follow-up), early complication rate (defined as the rate of complication within 30 days after surgery), and recurrent TR [defined as postoperative moderate and above TR (grade 2–4)].

### Risk Assessment of Bias in Included Studies

The risk of bias in the included studies was referenced to the Newcastle–Ottawa Scale (NOS). Evaluation items included the following: (1) representativeness of the exposed cohort, (2) selection of the non-exposed cohort, (3) ascertainment of exposure, (4) outcome of interest not present at the start of the study, (5) comparability of cohorts on the basis of the design or analysis, (6) assessment of outcome, (7) long enough follow-up for outcomes to occur, and (8) adequacy of follow-up of cohorts (34). Among them, the fifth item is 2 points, and the remaining 7 are 1 point. The score of the scale is 0–9, and when the score is ≥7, it is considered to be a study with a low risk of bias (35). The risk deviation assessment was completed by two authors independently, and when differences arose, they were resolved through discussion or negotiated by a third author until agreement was reached.

### Statistical Analysis

All data analyses were performed using RevMan5.3 software and Stata16. We chose unadjusted raw data because various studies did not adjust for the same set of confounding factors. Binary variables are represented by odds ratios (*OR*s), continuous variables are represented by mean differences (MDs) for consistent measurement units, and standardized mean differences (SMDs) are used for inconsistent measurement units. All variables were calculated with 95% *CI*s. All reported values of *p* are two-sided, and *p* < 0.05 was considered statistically significant. Heterogeneity tests were performed on the included studies using the *Q*-test and *I*^2^ test. The fixed effect model was used for analysis only when *p* > 0.10 and *I*^2^ ≤ 50%. Otherwise, the heterogeneity of the study was considered significant, and the random effects model (D-L method) was used for analysis.

## Results

### Characteristics of the Included Studies

**Figure 2:**
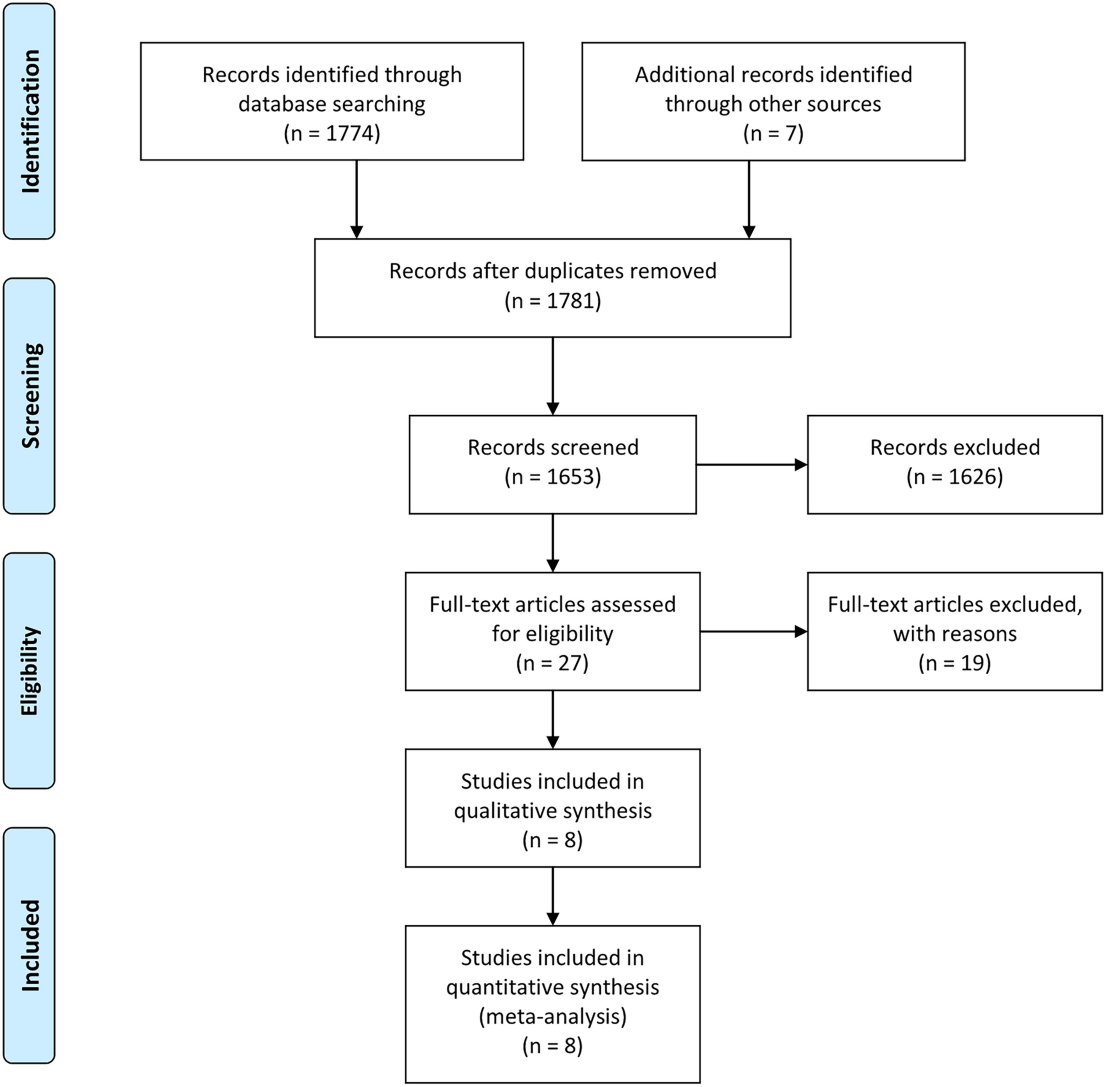
Flowchart of study selection for the present study.

The literature search identified 1,781 studies, and 1,653 remained after deleting duplicates. By reading the titles and abstracts, 1,626 irrelevant documents were eliminated, and the remaining 27 documents were read in full. Among them, 19 articles were excluded because they did not involve the 3D rigid ring. Finally, eight studies (36–43) that met the inclusion criteria were included in our systematic review and meta-analysis (as shown in Figure 2). All the included studies were retrospective cohort studies, of which five studies (36–40) compared 3D rigid ring annuloplasty with flexible band annuloplasty (flexible band group) and four studies (39, 41–43) compared 3D rigid ring annuloplasty with suture annuloplasty (suture group). The types of 3D rigid rings were all Edwards MC3, and the suture group all underwent De Vega annuloplasty. The severity of TR was evaluated using an apical four-chamber view and graded from 0 to 4+ (0: none, 1+: mild, 2+: moderate, 3+: moderate-to-severe, and 4+: severe). Two studies (38, 43) were adjusted using propensity-score matched (PSM) analysis. Table 1 summarizes the characteristics of all included articles. Table 2 shows the risk of bias results of the eight included studies.

**Table 1:** Baseline characteristics of individual studies.

**References**	**Year**	**Country**	**Period**	**Total (*n*)**	**3D-Rigid (*n*)**	**Control (*n*)**	**Type of control group**		**Follow-Up(month)**	
									**3D-Rigid**	**Control**
**3D-rigid vs. flexible**										
Ito et al. (36)	2017	Japan	2006.4–2015.4	98	41	57	Tailor flexible ring		65.6 ± 21.6	34 ± 12.8
Izutani et al. (37)	2010	Japan	2005.5–2007.12	117	82	35	Cosgrove-Edwards		21 ± 7	34.6 ± 9
Lee et al. (38)	2017	South Korea	2001.1–2012.12	581	211	370	Duran AnCore		28 (20–42)	71 (36–100)
Lin et al. (39)	2014	China	2006.1–2011.6	157	59	98	Duran ring or cosgrove band		39.6 (6–66)	39.6 (6–66)
Wang et al. (40)	2016	China	2009.9–2013.12	106	60	46	Cosgrove-Edwards		34.5 ± 9.3	34.5 ± 9.3
**3D-rigid vs. suture**										
Hou et al. (41)	2017	China	2012.1–2015.1	85	40	45	DeVega		32 (8–45)	32 (8–45)
Jiang et al. (42)	2019	China	2010.12–2012.12	69	35	34	DeVega		24	24
Lin et al. (39)	2014	China	2006.1–2011.6	301	59	242	Traditional or modified DeVega		39.6 (6–66)	39.6 (6–66)
Sohn et al. (43)	2019	South Korea	2003.3–2017.3	435	204	231	DeVega		102 (53–141)	102 (53–141)
**References**	**Age (years)**		**Female gender**		**NYHA functional class**		**Pre-Operative TR grade**		**LVEF (mm)**	
	**3D-Rigid**	**Control**	**3D-Rigid**	**Control**	**3D-Rigid**	**Control**	**3D-Rigid**	**Control**	**3D-Rigid**	**Control**
**3D-rigid vs. flexible**										
Ito et al. (36)	67.6 ± 8.9	68.6 ± 10.2	22	30	2.44 ± 0.54	2.58 ± 0.72	1.56 ± 0.66	1.65 ± 1.02	/	/
Izutani et al. (37)	72.4 ± 10	72.6 ± 11	53	18	2.92 ± 0.8	3.08 ± 0.7	2.68 ± 0.7	2.8 ± 0.67	57.5 ± 13	56 ± 8
Lee et al. (38)	58.5 ± 12.7	54.4 ± 12.8	135	236	/	/	3 ± 0.91	3.28 ± 0.77	/	/
Lin et al. (39)	47.7 ± 16.3	46.1 ± 14.7	31	52	2.93 ± 0.78	2.9 ± 0.78	3 ± 0.86	3 ± 0.86	56.2 ± 5.9	53.4 ± 4.1
Wang et al. (40)	58 ± 4.8	56 ± 5.2	29	27	3.17 ± 0.67	3.15 ± 0.66	3.47 ± 0.31	3.31 ± 0.39	50 ± 7.6	51 ± 6.3
**3D-rigid vs. suture**										
Hou et al. (41)	52.7 ± 14.2	51.6 ± 13.5	18	20	2.7 ± 0.6	2.5 ± 0.7	/	/	52.2 ± 6.6	51.4 ± 6.7
Jiang et al. (42)	41.28 ± 11.13	43.45 ± 12.18	18	17	2.54 ± 0.5	2.59 ± 0.49	2.16 ± 1.45	2.15 ± 1.46	/	/
Lin et al. (39)	47.7 ± 16.3	46.2 ± 15.4	31	128	2.93 ± 0.78	2.9 ± 0.78	3 ± 0.86	3 ± 0.86	56.2 ± 5.9	55.1 ± 5.9
Sohn et al. (43)	58.2 ± 12.9	60.6 ± 11.1	127	146	/	/	1.46 ± 1.03	2.03 ± 1.09	55.4 ± 8.9	57.3 ± 8.5

*3D, Three-Dimensional; NYHA, New York heart association; TR, Tricuspid regurgitation; LVEF, Left ventricular ejection fractions*.

**Table 2:** Results of NOS included in the study.

**Inclusion study**	**Selection**				**Comparability**	**Outcome**			**Total (minutes)**
	**①**	**②**	**③**	**④**	**⑤**	**⑥**	**⑦**	**⑧**	
Ito et al. (36)	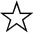	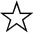	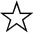	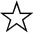	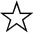	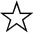	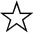	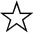	8
Wang et al. (40)	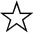	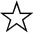	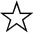	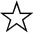	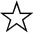	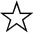	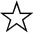	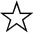	8
Lin et al. (39)	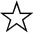	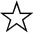	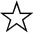	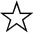	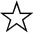	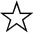	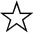	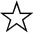	8
Lee et al. (38)	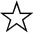	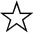	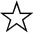	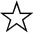	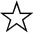 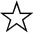	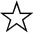	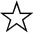	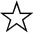	9
Izutani et al. (37)	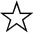	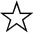	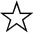	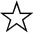	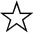	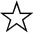	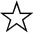	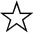	8
Jiang et al. (42)	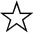	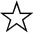	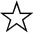	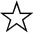	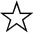	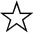	/	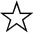	7
Sohn et al. (43)	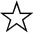	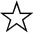	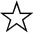	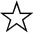	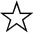 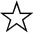	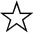	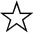	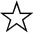	9
Hou et al. (41)	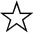	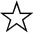	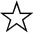	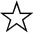	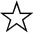	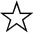	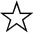	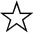	8

① *Representativeness of the Exposed Cohort* ② *Selection of the Non-Exposed Cohort* ③ *Ascertainment of Exposure Demonstration* ④*That Outcome of Interest Was Not Present at Start of Study* ⑤ *Comparability of Cohorts on the Basis of the Design or Analysis* ⑥ *Assessment of Outcome* ⑦ *Was Follow-Up Long Enough for Outcomes to Occur* ⑧ *Adequacy of Follow Up of Cohorts. The symbol 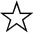 indicates one point and the symbol 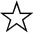 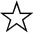 indicates two points*.

### Baseline Characteristic Analysis

**Figure 3:**
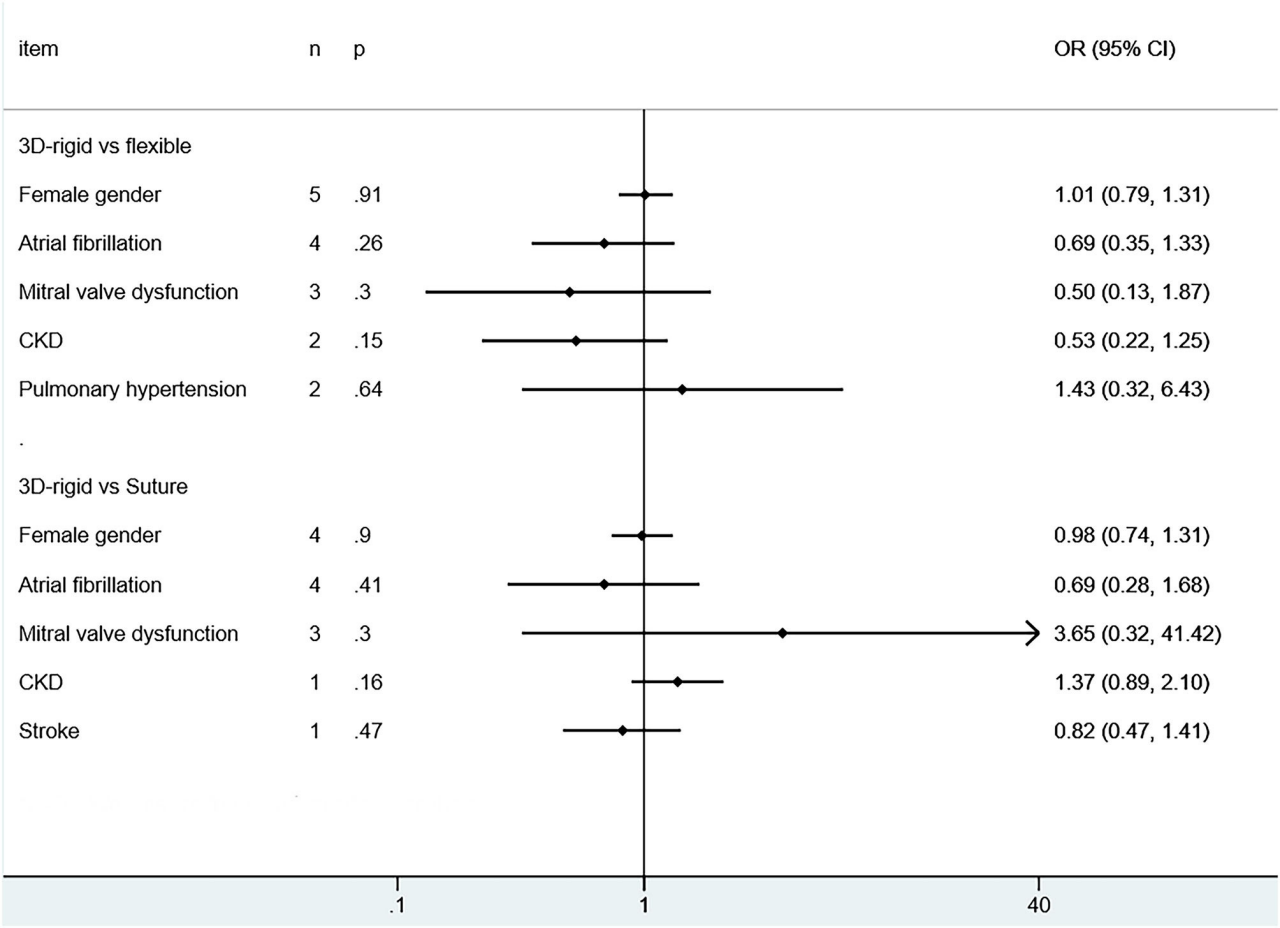
Pooled baseline characteristics and dichotomous variables.

**Figure 4:**
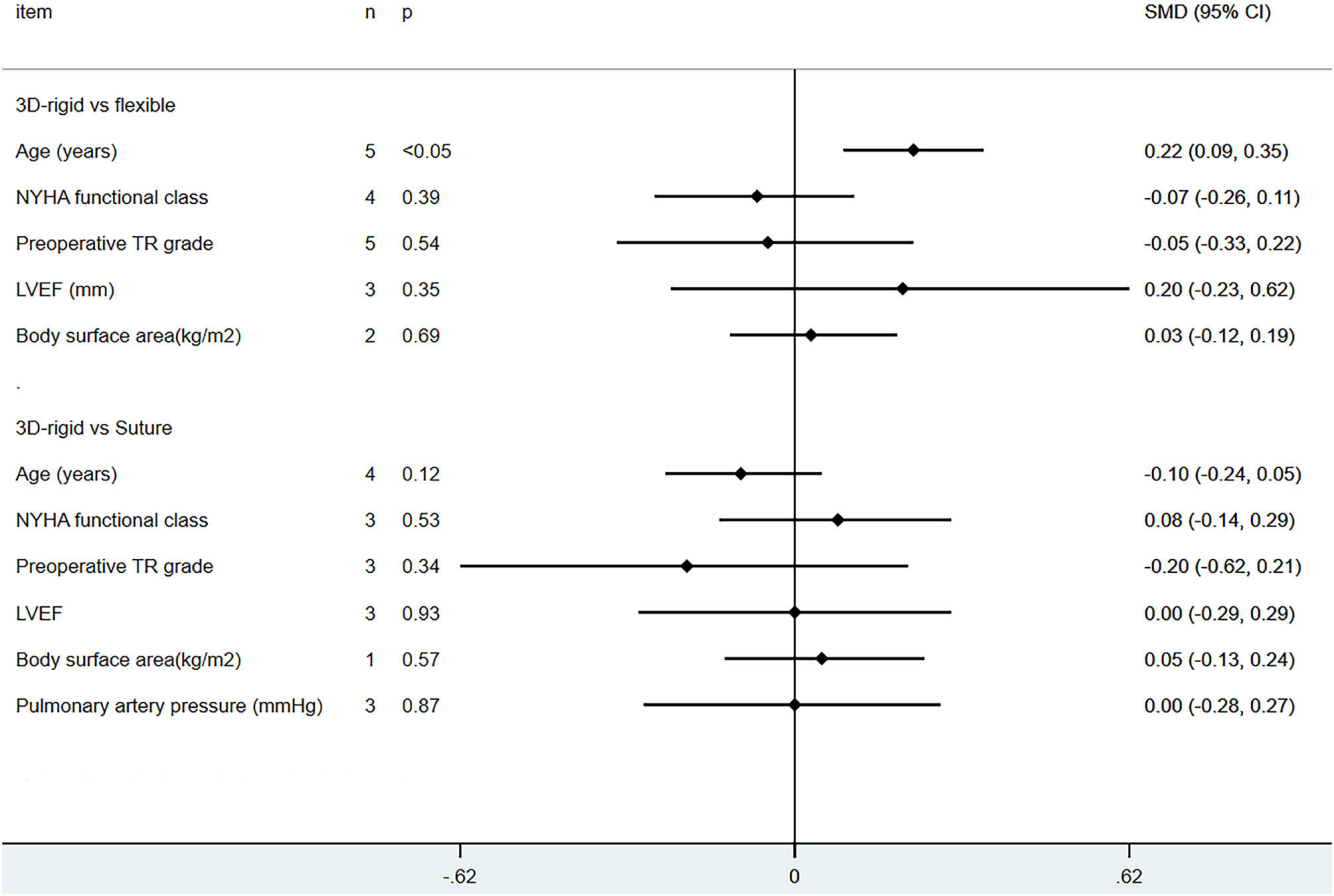
Pooled baseline characteristics and continuous variables.

Wang et al. (40) did not report whether it was combined with atrial fibrillation, and patients from other studies in both groups had atrial fibrillation. The vast majority of patients had moderate to severe TR (TR ≥ 2). Only in the flexible band group were patients with 3D rigid rings older than those with flexible bands [SMD = 0.22; 95% *CI* (0.09, 0.35); *p* < 0.05], and there was no difference in other baseline information. The baseline information was comparable (as shown in Figures 3, 4).

### Perioperative Characteristics and Outcomes

#### 3D Rigid Ring vs. Flexible Band

**Figure 5:**
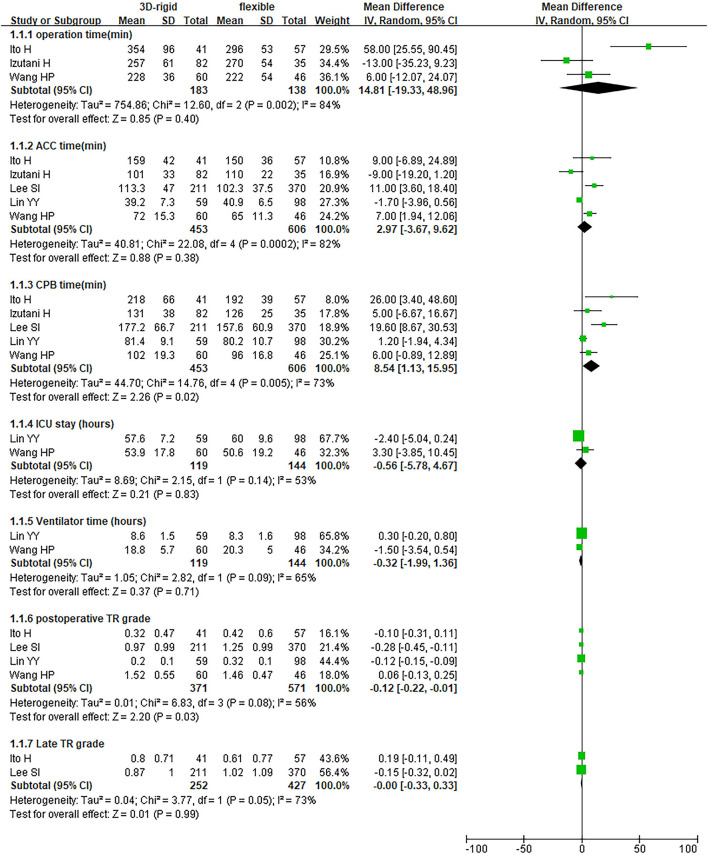
Meta-analysis of 3D rigid ring and flexible band annuloplasty, continuous variable, and random effects model.

**Figure 6:**
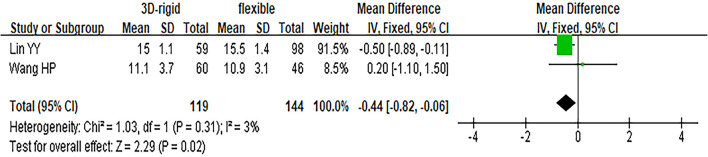
Meta-analysis of 3D rigid ring and flexible band annuloplasty, continuous variable, fixed effects model.

**Figure 7:**
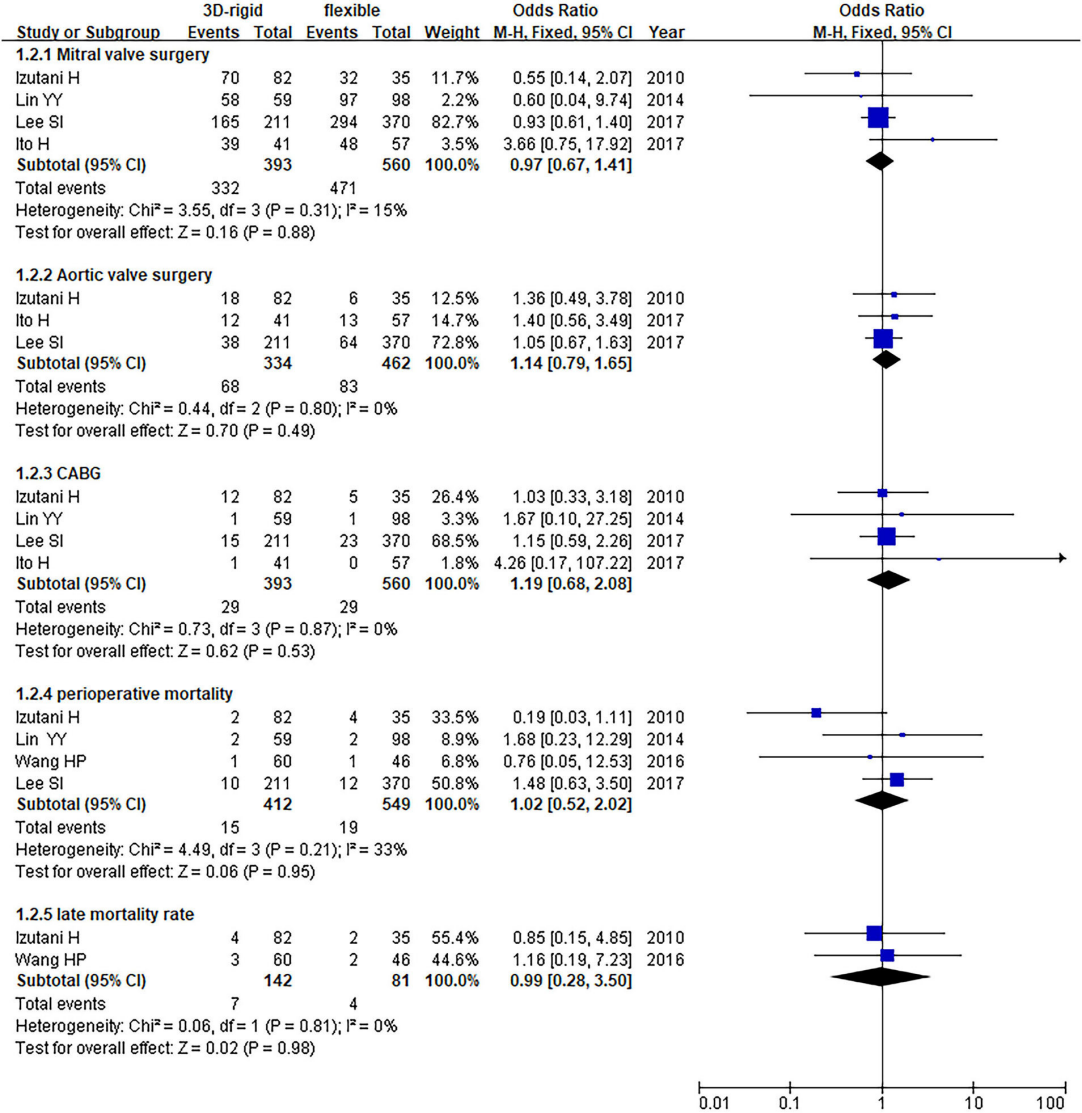
Meta-analysis of 3D rigid ring and flexible band annuloplasty, dichotomous variables, and fixed effects model.

**Figure 8:**
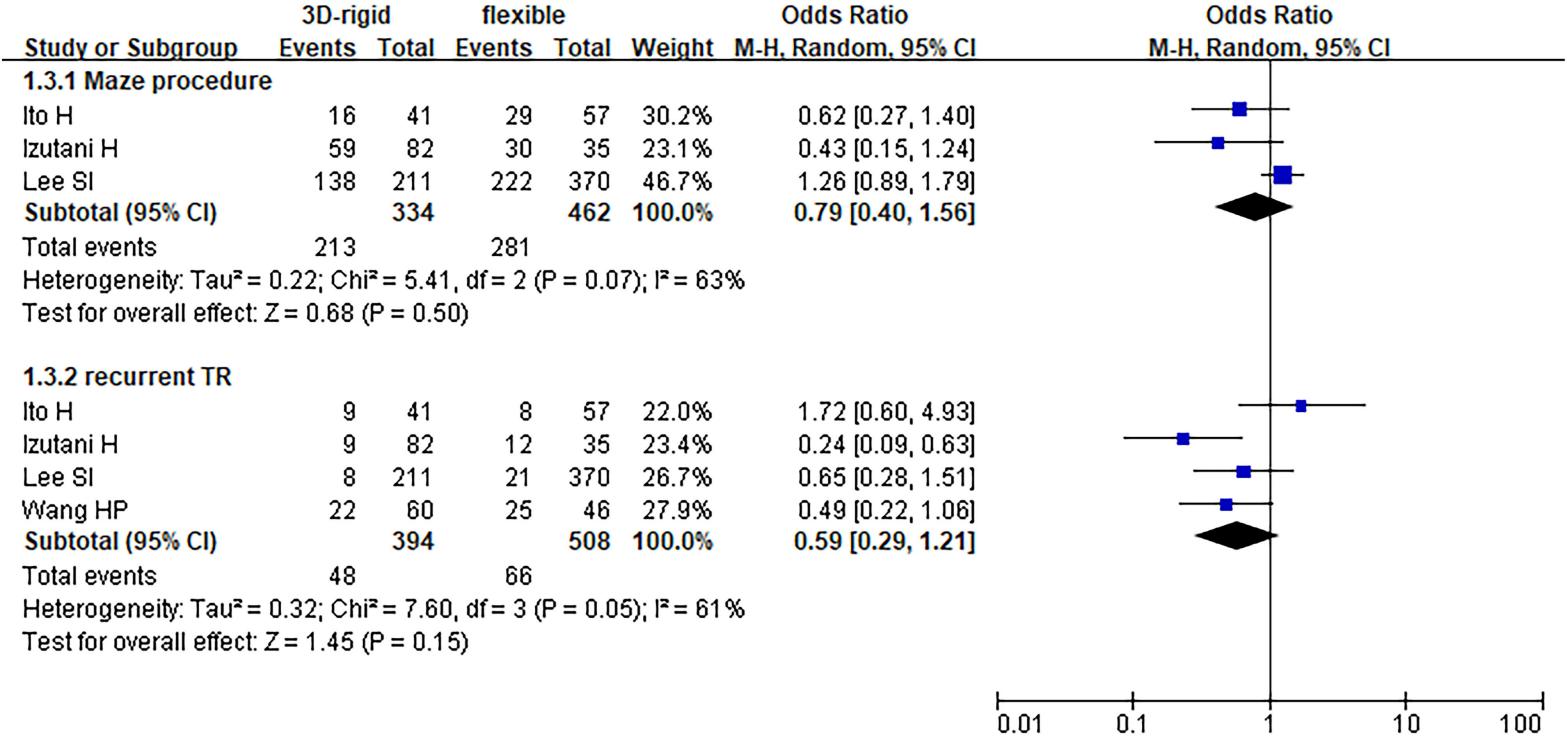
Meta-analysis of 3D rigid ring and flexible band annuloplasty, dichotomous variables, and random effects model.

In this group, 453 patients underwent 3D rigid ring annuloplasty, and 606 patients underwent flexible band annuloplasty. In the two groups of 3D rigid rings and flexible bands, there was no significant difference in operation time [mean difference (MD) = 14.81; 95% *CI* (−19.33, 48.96); *p* = 0.4]. Compared with the flexible band, the CPB time of the 3D rigid ring was longer, with an average of 8.54 min longer [MD = 8.54; 95% *CI* (1.13, 15.95); *p* = 0.02]. However, the hospital stay of the rigid 3D ring was 0.44 days shorter than that of the flexible band [MD = −0.44; 95% *CI* (−0.82, −0.06); *p* = 0.02]. There was no discrepancy in aortic cross-clamp time [MD = 2.97; 95% *CI* (−3.67, 9.62); *p* = 0.38], intensive care unit (ICU) stay [MD = −0.56; 95% *CI* (−5.78, 4.67); *p* = 0.83] or ventilator time [MD = −0.32; 95% *CI* (−1.99, 1.36); *p* = 0.71]. The main heart surgeries included mitral valve surgery [*OR* = 0.97; 95% *CI* (0.67, 1.41); *p* = 0.88], aortic valve surgery [*OR* = 1.14; 95% *CI* (0.79, 1.65); *p* = 0.49], coronary artery bypass grafting (CABG) [*OR* = 1.19; 95% *CI* (0.68, 2.08); *p* = 0.53], and maze procedure [*OR* = 0.79; 95% *CI* (0.40, 1.56); *p* = 0.50], but the results were not statistically obvious. There was no diversity in combined surgery between the two groups. The results are shown in Figures 5–8.

Regarding perioperative mortality [*OR* = 1.02; 95% *CI* (0.52, 2.02); *p* = 0.95] and late mortality [*OR* = 0.99; 95% *CI* (0.28, 3.50); *p* = 0.98], the results were not statistically significant. The postoperative TR grade of 3D rigid ring annuloplasty was 0.12 lower than that of flexible band annuloplasty [MD = −0.12; 95% *CI* (−0.22, −0.01); *p* = 0.03], and the 3D rigid postoperative treatment effect was better. However, there was no divergence in the TR level at the last follow-up [MD = 0; 95% *CI* (−0.33, 0.33); *p* = 0.99]. Early complications mainly included bleeding requiring surgery, low cardiac output syndrome, acute kidney injury, stroke, and arrhythmia requiring pacemakers, and the above indicators were not statistically significant. During the follow-up, there was no great discrepancy in recurrent TR [*OR* = 0.59; 95% *CI* (0.29, 1.21); *p* = 0.15] (as shown in Figures 5–8).

#### 3D Rigid Ring vs. Suture

**Figure 9:**
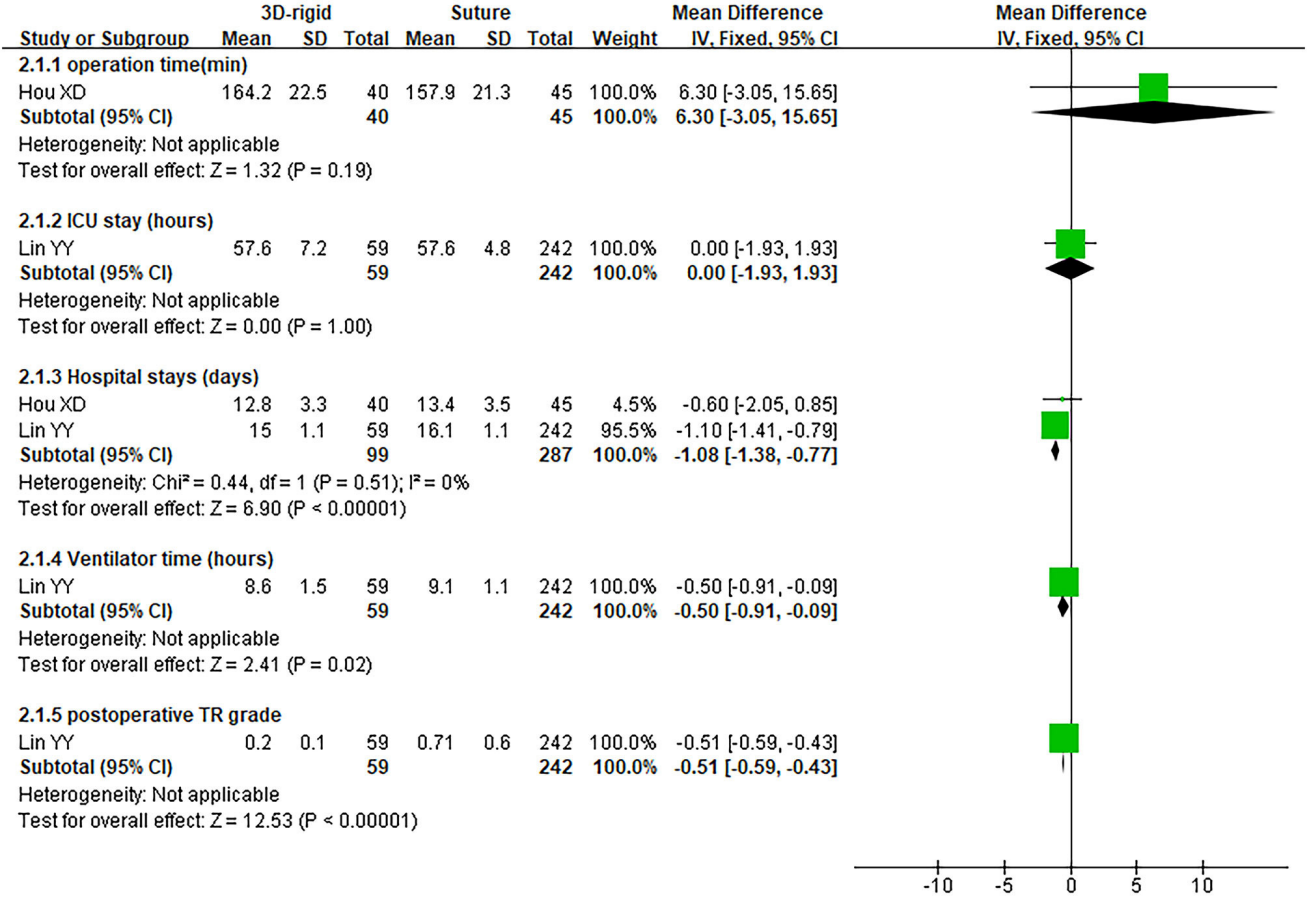
Meta-analysis of 3D rigid ring and suture annuloplasty, continuous variable, and fixed effects model.

**Figure 10:**
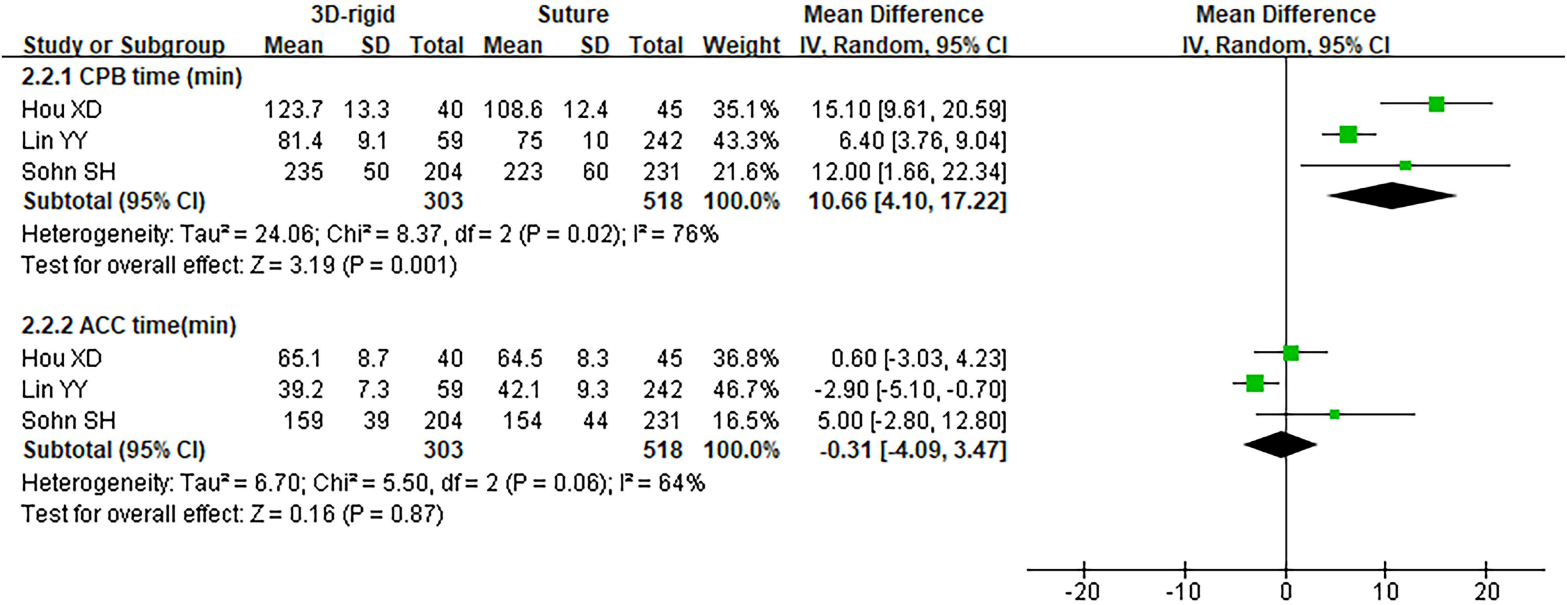
Meta-analysis of 3D rigid ring and suture annuloplasty, continuous variable, and random effects model.

**Figure 11:**
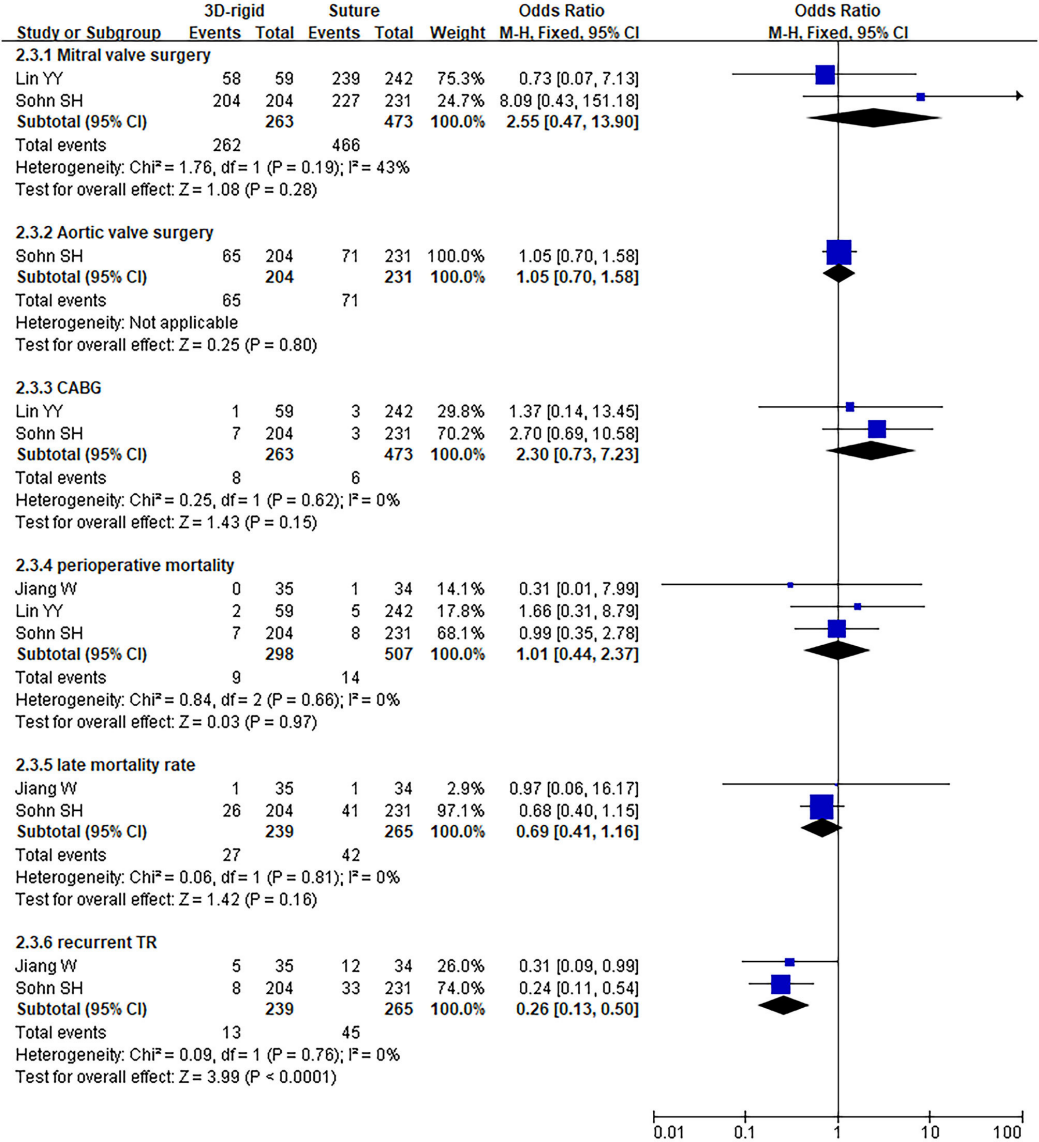
Meta-analysis of 3D rigid ring and suture annuloplasty, dichotomous variables, and fixed effects model.

**Figure 12:**
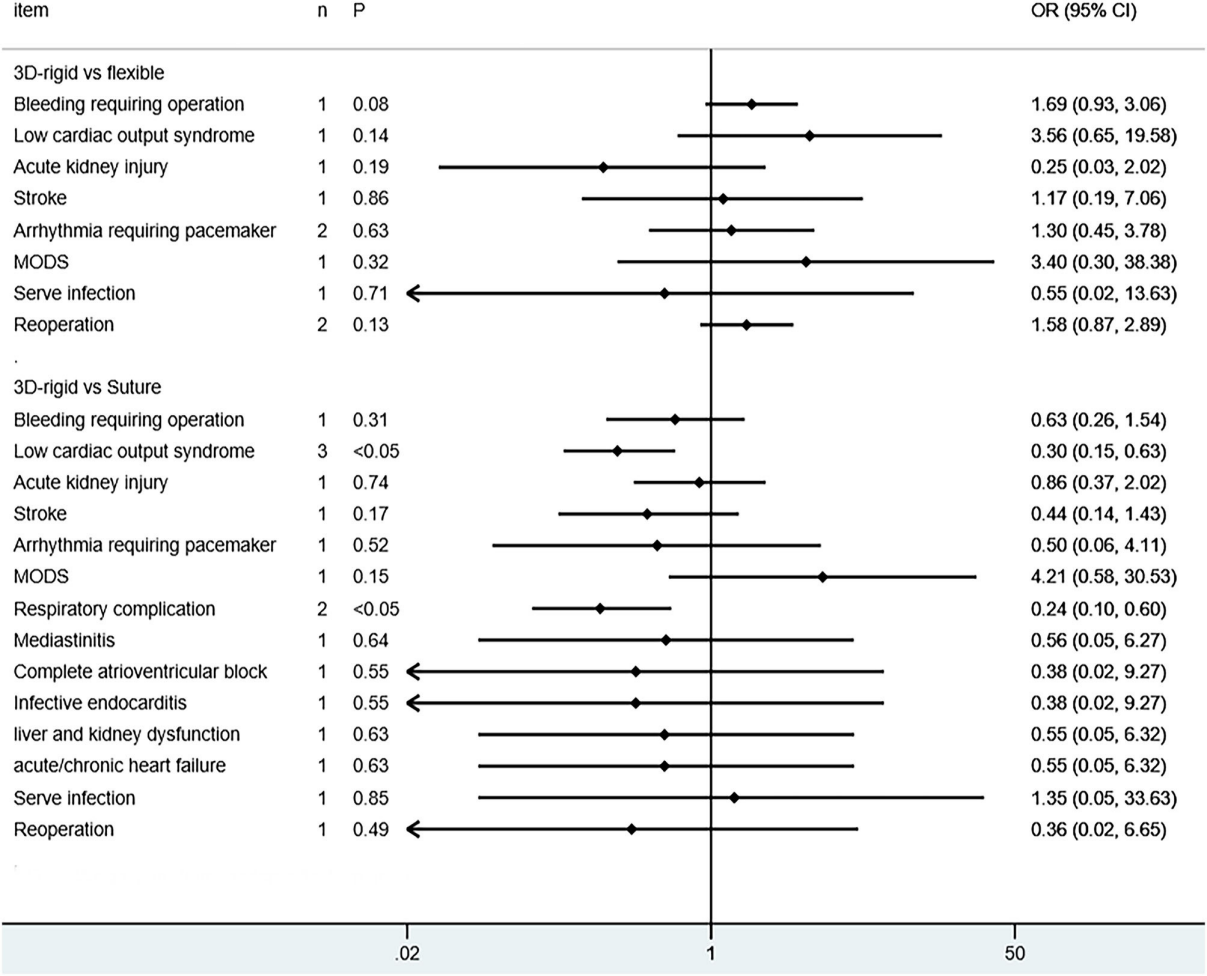
Meta-analysis of complications in the two groups.

In this group, 338 patients underwent 3D rigid ring annuloplasty, and 552 patients underwent suture annuloplasty. Most patients underwent other heart surgeries at the same time. The main heart surgeries included mitral valve surgery [*OR* = 2.55; 95% *CI* (0.47, 13.90); *p* = 0.28], aortic valve surgery [*OR* = 1.05; 95% *CI* (0.70, 1.58); *p* = 0.80], and CABG [*OR* = 2.30; 95% *CI* (0.73, 7.23); *p* = 0.15], and the results were not statistically significant. There was no difference in the combined cardiac surgery between the two groups. Compared with the suture group, the CPB time of the 3D rigid ring group was 10.66 min longer on average [MD = 10.66; 95% *CI* (4.10, 17.22); *p* = 0.001]. However, the hospital stays of the 3D rigid ring group were 1.08 days shorter than those of the suture group, and the ventilator time was 0.5 h shorter. There was no evident diversity in ACC time [MD = −0.31; 95% *CI* (−4.09, 3.47); *p* = 0.87], ICU stay [MD = 0; 95% *CI* (−1.93, 1.93); *p* = 1], perioperative mortality [*OR* = 1.01; 95% *CI* (0.44, 2.37); *p* = 0.97] or late mortality [*OR* = 0.69; 95% *CI* (0.41, 1.16); *p* = 0.16] between the two groups. The results are summarized in Figures 9–12.

The postoperative TR grade of 3D rigid ring annuloplasty was 0.51 lower than that of suture annuloplasty [MD = −0.51; 95% *CI* (−0.59, −0.43); *p* < 0.05]. Within the 5-year follow-up, patients who underwent 3D rigid ring annuloplasty had a lower TR recurrence than those who underwent suture annuloplasty [*OR* = 0.26; 95% *CI* (0.13, 0.50); *p* < 0.05]. The early incidence of low cardiac output syndrome [*OR* = 0.30; 95% *CI* (0.15, 0.63); *p* < 0.05] and respiratory complications [*OR* = 0.30; 95% *CI* (0.15, 0.63); *p* < 0.05] in the 3D rigid ring group was lower than that of suture annuloplasty, and there was little divergence in other early complications (as shown in Figures 9–12).

## Discussion

Pathological TR is more often secondary due to right ventricle (RV) dysfunction following pressure and/or volume overload in the presence of structurally normal leaflets (44). A large number of studies have found that the incidence of TR is high after cardiac surgery. In a study by Yilmaz et al. (45), progression of TR occurred in 67% of patients with rheumatic mitral valve (MV) disease after MV replacement (15) and in 74% of patients with ischemic mitral regurgitation (MR) after MV repair (46). In a study assessing the progression of TR in patients undergoing MV repair for functional MR in dilated cardiomyopathy, 18% of patients without TVA developed late TR progression. Patients with severe TR after MV replacement usually have a poor outcome after TV surgery. The perioperative mortality rate is 11–20%, even as high as 50% (47, 48). The tricuspid annulus and the mitral annulus are very close in anatomical position, and the movement of the tricuspid annulus may be affected by the mitral annulus (49). Studies have suggested that the imbalance of cardiac fiber skeleton stability may be an initiating factor of TR. After MV operation, although the artificial valve is still “normally” opened and closed, the fibrous skeleton between the MV device and the TV device has difficulty in maintaining the stability due to the destruction of cardiac biomechanics. The relatively strong movement of the TV device causes the annulus to gradually expand, which may eventually lead to TR (50). Tricuspid annuloplasty is accepted as the standard technique for correcting TR. However, the clinical choice of tricuspid annuloplasty has not yet been determined.

This study is a systematic review and meta-analysis to explore the efficacy of 3D rigid ring annuloplasty and other TAPs for the treatment of tricuspid regurgitation. We used the 3D rigid ring as an independent treatment method, summarized the current research status of the 3D rigid ring, and provided a reference for the clinical selection of an appropriate TAP.

In this meta-analysis, the results of the suture group collected from four studies showed that the post-operative TR grade of 3D rigid ring annuloplasty was 0.51 lower than that of suture annuloplasty, and the incidence of early postoperative complications was also lower. The 3D rigid ring showed early advantages. Compared with suture annuloplasty, the recurrent TR of 3D rigid rings within 5 years was significantly reduced, which indicated the advantages of 3D rigid rings over sutures. A meta-analysis published by Parolari A (26) in 2014 demonstrated that the all-cause mortality of ring and suture annuloplasty was similar, but the recurrent TR of ring annuloplasty remained lower, which is consistent with the results of our meta-analysis. In fact, there have been a large number of clinical studies exploring the ring and suture annuloplasty, and most of the studies supported that ring annuloplasty was better than suture. Therefore, ring annuloplasty is considered to be the standard technique for correcting TR and is widely used in clinical practice (25, 27, 28, 51). The results of this meta-analysis support 3D rigid ring annuloplasty, which has better curative effects than suture and can bring ideal early results to patients.

Over the years, ring annuloplasty has designed and implemented three types of equipment, such as soft flexible bands, standard rigid rings, and 3D rigid rings (52). The results of studies comparing the flexible band and the rigid ring (53, 54) proclaimed that both have clinically acceptable early and late mortality, and compared with the rigid ring, the total TR of the flexible band was higher. However, a network meta-analysis conducted by Yokoyama (55) did not observe a difference in the recurrence rate of TR between the two types of annuloplasty. The results indicated that there was no significant difference between the flexible band and the rigid ring approaches. We believe that these two contradictory results may be related to the spatial structure of the rigid ring, and it is necessary to explore the 3D rigid ring approach separately. Filsoufi et al. (56) suggested using a 3D rigid ring, believing that it has a three-dimensional structure, is easy to implant, and can significantly reduce FTR. Additionally, there are studies showing that the flexible band that could keep the tricuspid valve annulus in good physiological movement during the cardiac cycle is not easy to split and has an excellent effect (57). In this meta-analysis, the results of the flexible band group from five studies showed that the postoperative TR grade of 3D rigid ring annuloplasty was 0.12 lower than that of the flexible band overall, while the operation time was longer, with an average of 8.54 min longer. Apart from this, there were no significant differences between the other results. In other words, the 3D rigid ring was only slightly stronger than the flexible band in reducing the TR ability, and there was no advantage in other aspects. The results of our research did not support the claim that the 3D rigid ring was superior to the flexible band due to the three-dimensional structure. In addition, considering the economic burden of patients, the cost of 3D rigid ring annuloplasty is generally higher than that of flexible band annuloplasty, while the early and long-term effects of the two procedures are similar to those of patients. It seems that a flexible band should be recommended to patients.

We searched all the available literature, and we did not find studies comparing the 3D rigid ring annuloplasty with Kay annuloplasty. Jung et al. (58) compared the early efficacy of Edwards MC3 annuloplasty ring and Tri-Ad Adams tricuspid ring (Tri-Ad; Medtronic, Minneapolis, MN, USA) in repairing the tricuspid valve in 2018, and its results indicated that the two have similar effects in the treatment of TR. The Tri-Ad ring, which consists of a large open area to protect the conduction system and flexible ends combined with a 3D semirigid midportion could not be categorized, so it was not included.

In clinical practice, treating or interfering with TV insufficiency has become an inevitable procedure after left-sided heart surgery because the progression of TR increases postoperative mortality and ultimately defeats the purpose of corrective heart surgery. Significant TR occurring late after left heart surgery is observed in up to 40% of patients, with a median survival of 5 years (59). An increase of >2 grades in TR with respect to preoperative echocardiography is reported in ~50% of patients who undergo isolated MV repair (15, 60). The latest ESC/EACTS (17) and ACC/AHA (61) guidelines both agree that patients undergoing left valve surgery should be treated for severe TR. For patients with mild or moderate TR with a dilated annulus (ECG ≥ 40 or >21 mm/m^2^), tricuspid annuloplasty should be considered at the same time. A recent meta-analysis (62) supports this view, which shows that patients with mild to moderate TR undergoing tricuspid annuloplasty during MV repair can significantly reduce all-cause mortality and cardiovascular death. Safe and effective TAP is the focal point for the effective treatment of heart valve disease.

Observational studies have compared different TAPs, but no randomized trials have yet been conducted. Although most surgeons currently believe that the 3D rigid ring annuloplasty is more durable and is associated with better long-term and event-free survival, there is still a lack of high-quality evidence (20, 63). This systematic review explored the efficacy of 3D rigid ring annuloplasty and other tricuspid annuloplasties in the treatment of TR. Compared with sutures, the 3D rigid ring holds obvious advantages. Its early effect is better than that of sutures, and it has an acceptable early mortality rate. Both 3D rigid rings and flexible bands have good short-term effects in the treatment of TR, but the long-term effects are still uncertain. Obviously, it is necessary to further study the long-term effects of 3D rigid ring annuloplasty on TR. In addition, according to the evidence we obtained, the 3D rigid ring and the flexible band have similar effects. One of the influencing factors may be the lack of attention to TR classification in the included studies. Patients with mild, moderate, and severe TR were uniformly analyzed. Future research should consider mild/moderate TR or only tricuspid annulus expansion without TR as the research focus and explore whether the TV should be treated at the same time when left heart valve surgery is combined with the above conditions. If these issues are dealt with, then the question of what measures should be recommended should be addressed.

In recent years, TTVI has gradually developed to treat TR, and several TTVI devices have also appeared, such as the Mitra Clip NT system (64). However, these technologies are still in the development stage, and there is no evidence that percutaneous intervention for advanced TR is beneficial. Therefore, a systematic evaluation of tricuspid valvuloplasty is necessary. This meta-analysis summarizes the current status of the 3D rigid ring and provides clinical information for the surgical treatment of TR for TTVI. The 3D rigid ring annuloplasty may be used as a benchmark for evaluating the performance of TTVI to explore whether the efficacy of TTVI is comparable with that of tricuspid annuloplasty, which may be an important direction for future research on TR.

## Limitation

Our research also has some limitations. Therefore, a large number of clinical studies are all case reports, and there is little evidence for comparison between TAPs, which leads to insufficient sample size and affects the potency of the results. Furthermore, all included studies were retrospective observational studies. Their follow-up time was inconsistent, which may have selection bias and data aggregation bias. Some of the included studies did not conduct a detailed follow-up on the postoperative status of patients; for example, the long-term TR grade and New York Heart Association (NYHA) grade of patients were not reported, which led to the inability to know whether the patients benefited from the operation. Finally, the studies report fewer long-term results whose differences may not be detected. Considering these limitations, the results of this study should be interpreted carefully. In future studies, detailed follow-up information should be collected. As a minimum requirement, the NYHA grade should be proposed or displayed during follow-up. Long-term follow-up studies are needed to verify the findings of this study in the future.

## Conclusions

Compared with suture annuloplasty, the postoperative TR grade and the recurrent TR of 3D rigid ring annuloplasty are significantly reduced, which has early advantages. The 3D rigid rings provide an acceptable short-term effect similar to that of the flexible bands, and a significant difference between the approaches is not observed. Although 3D rigid rings have been widely favored in clinical applications, the existing evidence shows that they do not present significant advantages. This conclusion is based on the limited, short-term data available at the time of the study. Further research is clearly needed on the long-term effects of 3D rigid ring annuloplasty for TR.

## Data Availability Statement

The original contributions presented in the study are included in the article/supplementary material, further inquiries can be directed to the corresponding author/s.

## Author Contributions

KY, Y-HM, and TY: conceptualization and validation. Y-HM, WW, J-GX, and JG: data collection. TY, Y-HM, S-EH, X-MX, and MJ: formal analysis. TY: funding acquisition. TY, Y-HM, JG, S-EH, and MJ: investigation. KY, Y-HM, WW, and TY: methodology. TY and Y-HM: software, supervision, and writing—original draft. TY, Y-HM, KY, JG, J-GX, X-MX, S-EH, WW, and MJ: writing—review and editing. All authors contributed to the article and approved the submitted version.

## Funding

This work was funded by the Natural Science Foundation of Gansu Province (21JR1RA027) and the Health Industry Scientific Research Project of Gansu Province (GSWSKY2016-04).

## Conflict of Interest

The authors declare that the research was conducted in the absence of any commercial or financial relationships that could be construed as a potential conflict of interest.

## Publisher's Note

All claims expressed in this article are solely those of the authors and do not necessarily represent those of their affiliated organizations, or those of the publisher, the editors and the reviewers. Any product that may be evaluated in this article, or claim that may be made by its manufacturer, is not guaranteed or endorsed by the publisher.
